# Associations between maternal dietary patterns and infant birth weight, small and large for gestational age in the Norwegian Mother and Child Cohort Study

**DOI:** 10.1038/s41430-018-0356-y

**Published:** 2018-11-20

**Authors:** Linda Englund-Ögge, Anne Lise Brantsæter, Julius Juodakis, Margareta Haugen, Helle Margrete Meltzer, Bo Jacobsson, Verena Sengpiel

**Affiliations:** 1 Department of Obstetrics and Gynecology, Sahlgrenska University Hospital, Gothenburg, Sweden; 2 Department of Obstetrics and Gynecology, Sahlgrenska Academy, Gothenburg University, Gothenburg, Sweden; 3 Division of Infection Control and Environmental Health, Norwegian Institute of Public Health, Oslo, Norway; 4 Domain of Health Data and Digitalisation, Norwegian Institute of Public Health, Oslo, Norway

**Keywords:** Epidemiology, Nutrition, Epidemiology, Nutrition

## Abstract

**Background/Objectives:**

To assess whether quality of maternal diet affects birth weight and the risk of small for gestational age (SGA) and/or large for gestational age (LGA) babies.

**Subjects/Methods:**

This study is based on the Norwegian Mother and Child Cohort Study (MoBa) and includes 65,904 pregnant women who answered a validated food frequency questionnaire at mid-pregnancy. Three maternal dietary patterns were extracted based on characteristics of food items in each pattern. From these we created four non-overlapping groups: “high prudent,” “high Western,” “high traditional,” and “mixed”. We obtained information about birth weight from the Norwegian Medical Birth Registry and calculated birth weight *z*-scores, SGA, and LGA according to an ultrasound-based, population-based, and a customized growth standards. Associations were studied by linear and multiple logistic regression.

**Results:**

Compared to the high Western group, the high prudent group was associated with lower birth weight (*β*_ultrasound_ *z*-scores −0.041 (95% confidence interval (CI): −0.068, −0.013)) and the high traditional group with higher birth weight (*β*_ultrasound_ 0.067 (95% CI: 0.040, 0.094)) for all three growth standards. The high prudent pattern was associated with increased SGA risk (SGA_ultrasound_ odds ratio (OR) 1.25 (95% CI: 1.02, 1.54)) and decreased LGA risk (LGA_population_ OR 0.84 (95% CI: 0.75, 0.94)), while the high traditional group on the contrary was associated with decreased SGA (SGA_customized_ OR 0.92 (95% CI: 0.84, 0.99)) and increased LGA risk (LGA_population_ OR 1.12 (95% CI: 1.02, 1.24)).

**Conclusions:**

Food quality was associated with birth weight in this well-nourished Norwegian population. Food quality may affect a woman’s risk of giving birth to a SGA or LGA baby.

## Introduction

Abnormal infant birth weight is correlated with increased infant mortality and morbidity [1–4**]**. In about 20% of all births, the babies are born either small for gestational age (SGA) or large for gestational age (LGA) [5]. Intrauterine growth restriction increases the risk of intrauterine fetal death [1], hypoxia during delivery [6], neonatal hypoglycemia [7], and necrotizing enterocolitis, a severe intestinal infection [8]. LGA is associated with prolonged delivery, excessive maternal hemorrhage, severe vaginal ruptures, and cesarean sections [9]. Moreover, changes in utero can negatively impact the baby’s health later in life, increasing the risk, for instance, of diabetes and cardiovascular disease [10], according to the Developmental Origins of Health and Disease (DOHaD) principle [11–13].

Besides genetic potential [14] and placenta function [15], maternal nutritional status is the major contributor to birth weight [16], but the associations between *overall* diet quality and birth weight and the risks of SGA and LGA are not well understood. Several studies have focused on individual foods or nutrients in relation to birth weight [17, 18]. However, individual food items are consumed as part of a total and the effect of a single nutrient may be difficult to ascertain. In recent years, the focus has thus shifted towards analysis of dietary patterns. A data-driven approach enables better coverage of the *quality* and, to some extent also, the *quantity* of food [19**–**21], without any predefined patterns [22]. Some studies have shown that unhealthy dietary patterns increase the risk of babies being born SGA [23, 24], while others found no such association [25]. Dietary habits are strongly associated with a population’s culture, and contents of diet in a specific pattern. Therefore, the perception of “healthy” and “unhealthy” differs, making comparison between populations difficult. Comparison between different studies and application of results from one population to another is challenging but important as this might help to understand and interpret the findings. In addition to population differences, inconsistent results between studies might be due to the use of different outcome definitions, as there is no international consensus on growth standards, and SGA or LGA definition.

Information about how maternal food quality is correlated to unfavorable birth weight could provide a basis for nutritional interventions to improve both maternal and infant health. The aim of this study was to examine associations between data-driven maternal dietary patterns and birth weight, SGA and LGA, based respectively on an ultrasound-based, population-based, and a customized growth standard, in a population-based pregnancy cohort.

## Subjects and methods

### Population and study design

The Norwegian Mother and Child Cohort Study (MoBa) is a prospective, population-based pregnancy cohort study conducted by the Norwegian Institute of Public Health [26]. Participants were recruited from all over Norway between 1999 and 2008. The cohort includes 114,500 children and 95,200 mothers [27]. Pregnant women were invited to participate in the study by postal invitation in connection with their first routine ultrasound scan; 40.6% consented. Participating women were asked to answer questionnaires covering a wide range of information. Birth records from the Medical Birth Registry of Norway (MBRN) were linked to the MoBa dataset [26, 28]. MoBa has a license from the Data Inspectorate. This study is based on version V of the data files released for research in 2010 and was approved by the Regional Committee for Ethics in Medical Research (2010/2683/REK). Informed written consent was obtained from all women.

This study is based on two questionnaires, respectively, answered around gestational weeks 15 (Questionnaire 1) and 22 (Questionnaire 2). In Questionnaire 1, women reported, among other things, demographics, lifestyle, obstetric history (e.g., contraceptives, previous pregnancies), illnesses, health-related factors, physical activity, medications, and dietary supplements. In Questionnaire 2, they recorded average daily intake of food, beverages, and dietary supplements since the beginning of pregnancy. Questionnaire 2 is a semi-quantitative food frequency questionnaire (FFQ), developed and validated for use in MoBa [29]. To be eligible for this study, women had to have answered Questionnaires 1 and 2 and to have delivered a singleton, live baby. Women with any type of diabetes were excluded from the study. To avoid multiple dependent observations, only the first enrolled pregnancy was included if women had participated in MoBa during more than one pregnancy. Detailed inclusion criteria and a flow chart of inclusion have been presented in a previous study on the same population [30]. We additionally excluded 96 women due to improbable recorded birth weight, leaving 65,904 mother–child pairs for analysis and inclusion in the final dataset.

### Birth weight (*z*-score), SGA, and LGA definition

Information about birth weight and gestational age was obtained from the MBRN.

Since there is no consensus on growth standards, we assessed birth weight, SGA, and LGA according to the following three approaches:

1. SGA/LGA, ultrasound-based: >2 standard deviation (SD) above or below the expected birth weight for any given gestational age, according to Marsal’s ultrasound-derived growth curves [31].
2. SGA/LGA, population-based: <10th or >90th percentile, according to gestational age-based growth curves derived from the Norwegian newborn population [32].
3. SGA/LGA, customized: <10th or >90th percentile, as suggested by Gardosi et al. [33], is based on Hadlock et al.’s [34] ultrasound-derived growth curves and takes into account additional characteristics such as infant sex and maternal weight, height, and parity [35]. Infant birth weight *z*-scores were calculated according to the growth standards described above.

### Dietary information

The MoBa FFQ was designed to cover maternal diet during the first 4–5 months of pregnancy. In a validation study with 119 MoBa participants, FFQ intakes were evaluated using four-day weighed food diaries and biological markers in blood and urine as reference measures [36]. The FFQ has been shown to correctly rank the MoBa pregnant women’s dietary intakes of energy, nutrients, and foods [29, 37–39]. The FFQs were optically read and food frequencies were converted into daily intakes of foods (g per day) and nutrients using FoodCalc and the Norwegian Food Composition Table [40]. A total of 255 food and beverage items were recorded with the FFQ. We aggregated similar food items into 58 non-overlapping food groups [41], which were used as input variables for extraction of the dietary patterns [30].

### Identification of non-overlapping dietary groups

We used a data-driven technique to empirically derive dietary patterns. Factor analysis with principal components is a technique used to reduce the dimension of the data by forming new linear combinations of the original observed variables based on correlation. Daily intakes (g per day) of foods and beverages in the 58 food groups were entered into the model and orthogonal (varimax) rotation was performed. Each participant is given scores on all factors (“patterns”), and the scores indicate the adherence of each individual to the respective dietary pattern. Based on visual examination of a scree plot, three dietary patterns were identified and respectively denoted prudent, Western, and traditional, based on the constituent foods. More details can be seen in Supplementary Table S1 and an earlier publication by our group [30]. We divided the pattern scores into tertiles and created non-overlapping dietary groups comprising individuals with the highest scores in each pattern. The new groups were named high prudent, high Western, and high traditional. To be included in the high prudent group, women had to be in the highest tertile of the prudent pattern and at the same time have scores in the middle or lowest tertile of the other two patterns. The same procedure was used for the high Western and high traditional groups. Women with pattern scores who did not fit into any of these predefined groups were classified as a mixed group. We used the high Western group as the reference category, to make the results comparable to a study by Knudsen et al. [24] based on the Danish National Birth Cohort, a similar cohort.

### Covariates

Nine covariates were chosen a priori because of known associations with birth weight: maternal age at delivery, maternal pre-pregnant body mass index (BMI), height, parity, smoking during pregnancy, alcohol intake, educational level, total energy intake, and household income. Data on maternal age was collected from the MBRN. Maternal age was used as a continuous variable. BMI was calculated from pre-pregnancy weight and height as reported in Questionnaire 1, and data were analyzed as categories (<18.5, 18.5–24.9, 25–29.9, and ≥30 kg/m^2^). Only women who reported weight between 35 and 180 kg and height above 1.40 m were included. Maternal height was divided into quartiles and used as a categorical variable (≤1.64, 1.65–1.68, 1.69–1.72, and ≥ 1.73 cm). Data on parity were collected from the MBRN as well as from MoBa and were divided into two categories: nulliparous or parous. Information about smoking habits during pregnancy was collected from Questionnaire 1 and the variable was categorized as: non-smoker, occasional, or daily smoker. Alcohol intake during pregnancy was analyzed as a dichotomous variable (yes/no). Maternal educational level was divided into three categories (≤12, 13–16, and ≥17 years of school). Total energy intake (kilojoules) was collected from the FFQ, and analyzed as a continuous variable. We collected information from Questionnaire 1 about income and the data were divided into three annual income categories: both partners’ <300,000 Norwegian Krone (NOK), participant’s or partner’s ≥300,000 NOK per year, and both partners’ ≥300,000 NOK. Physical activity level is known to be correlated to dietary patterns and obviously affects the net energy intake [42], which in turn affects fetal growth [43]. In the subanalysis, we also adjusted for leisure physical activity during pregnancy (four categories: none, less than once weekly, 1–2 times weekly, and ≥3 times weekly), reported in gestational week 17. We conducted sensitivity analyses to examine the potential impact of chronic diseases, including chronic hypertension, chronic kidney disease, rheumatoid arthritis, systemic lupus erythematosis, and sclerodermi, as dichotomous variables (yes/no), as well as eating disorders as these diseases can affect both food intake and birth weight [44**–**47]. Information on self-reported chronic diseases and current eating disorders was collected from Questionnaire 1.

### Statistical methods

PASW Statistics software version 19 for Windows (SPSS Inc., IBM Company, Chicago, IL, USA) was used for statistical analyses and *p* values (two sided) <0.05 were considered statistically significant. Linear models were produced in R 3.3.0 [48]. The four non-overlapping dietary groups were used as a four-category exposure variable in logistic regression. The high Western category was the reference category, and odds ratios (ORs) and 95% confidence intervals (CIs) for the high prudent, high traditional, and mixed patterns were calculated. Variables included in the adjusted models were: maternal age, height, BMI, parity, smoking, alcohol intake, total energy intake, and educational level, as well as household income. The associations between dietary groups and SGA/LGA were also examined after stratifying the study population according to BMI (<25 and ≥25 kg/m^2^). Further subanalyses were performed with additional adjustment for chronic diseases, eating disorders, and physical activity during pregnancy.

## Results

### Food characteristics within the dietary groups

Energy and nutrient intakes in the dietary groups reflected the quality and quantity of the food intakes (Table 1). Mean daily energy intake was highest in the high Western group, at 2434 kcal (10,231 kJ), and lowest in the high prudent group, at 2197 kcal (9241 kJ). Women in the high Western group also had the highest intake of fat, carbohydrates, and added sugar, and the lowest intake of dietary fiber, while women in the high prudent group had the opposite intakes for all these variables. Women in the high traditional group had the highest mean intake of protein. When comparing energy percent from macronutrients between the four groups, the high Western group had the highest percentage of total energy from fat, carbohydrates, and sugar, and the lowest energy percentage from protein. The high prudent group had the lowest percentage of energy from fat and sugar, and women in this group also had the highest intake of dietary fiber.

**Table 1:** Distribution of energy and macronutrients according to dietary groups

	High Western	High prudent	High traditional	Mixed
Energy (kcal)	2434^a^ ± 519^b^	2197 ± 466*	2227 ± 469*	2337 ± 700*^c^
Fat (g)	86 ± 20	74 ± 20*	77 ± 20*	81 ± 27*
Fat energy%^d^	32 ± 7.5	27 ± 7.2*	28 ± 7.2*	30 ± 10*
Protein (g)	84 ± 18	87 ± 18*	88 ± 19*	88 ± 24*
Protein energy%^d^	14 ± 3.1	15 ± 3.1*	15 ± 3.3*	15 ± 4.0*
Carbohydrate (g)	329 ± 85	295 ± 72*	296 ± 70*	313 ± 104*
Carbohydrate energy%^d^	56 ± 14	50 ± 12*	50 ± 12*	53 ± 18*
Dietary fiber (g)	28 ± 8.0	34 ± 10*	30 ± 8.2*	31 ± 12*
Dietary fiber g/MJ^d^	2.7 ± 0.6	3.7 ± 0.8*	3.2 ± 0.6*	3.2 ± 0.7*
Sugar (g)	86 ± 49	50 ± 24*	51 ± 27*	63 ± 40*
Sugar energy %^d^	15 ± 8.4	8.5 ± 4.1*	8.7 ± 4.6*	11 ± 6.7*

^a^Mean daily intake

^b^Standard deviation

^c^* *p* < 0.001 obtained by linear regression with high Western as the reference category

^d^The contribution to total energy intake by macronutrient. In the Nordic countries, the recommendations are 25–40% of total energy intake from fat, 10–20% from protein, 45–60% from carbohydrates, and <10% from sugar. For dietary fiber the recommendation is >3 g/MJ [72].

After ranking the pattern scores into tertiles and creating the four non-overlapping dietary groups as described above, the means and SDs for daily food intakes in the four groups clearly reflected the food items typical of the three original patterns (Supplementary Table 1). For instance, women in the high Western group had high intakes of sweet drinks, processed meat products, and refined cereal products, those in the high prudent group had high intakes of vegetables, fruit, and whole-grain cereal products, and those in the high traditional group had high intakes of lean fish, fish products, boiled potatoes, and cooked vegetables. Comparison of food group intakes between the four dietary groups showed that the high Western group had the highest intakes of energy-dense and ultra-processed foods (e.g., sugary drinks, French fries, and sweets). The high prudent group had the highest intakes of low-energy, nutrient-dense, and plant-based foods (e.g., vegetables and fruits), and the high traditional group had the highest intakes of Nordic foods (e.g., dark bread, low fat milk, lean fish, and boiled potatoes).

### Maternal characteristics by dietary groups

Women in the high Western group were youngest, had the highest BMI, were more often occasional or daily smokers, and were less educated compared to the other three groups (Table 2). Also, they had the lowest levels of physical activity at gestational week 17, but no significant differences in other chronic diseases compared to the other groups. In contrast, women in the high prudent group were oldest, had the lowest BMI, were less often smokers, and had the lowest reported intake of alcohol during pregnancy. They also had the highest educational level, highest household income, and highest level of physical activity reported in gestational week 17. Nausea during pregnancy was less common, but the highest percentage of women with eating disorders was found in the high prudent group. Women in the high traditional group had similar characteristics as women in the mixed group, for example, proportions of smokers, users of alcohol during pregnancy, educational level, and level of physical activity.

**Table 2:** Maternal characteristics in the four non-overlapping dietary groups in 65,904 mother–infant pairs in the Norwegian Mother and Child Cohort Study

	High Western	High prudent	High traditional	Mixed	*p* value
*n* (%)	9562 (14)	10,150 (15)	9754 (15)	36,438 (55)	
Maternal age at delivery (years)	28.7 ± 4.5^a^	31.3 ± 4.1	30.3 ± 4.8	30.0 ± 4.6	<0.001^b^
Pre-pregnancy body mass index (kg/m^2^*)*	24.6 ± 4.6	23.1 ± 3.6	24.1 ± 4.2	24.0 ± 4.2	<0.001^b^
Birthweight (g)	3549 ± 577	3503 ± 542	3605 ± 561	3552 ± 553	<0.001^b^
Parity					
Nulliparous	5039 (52.7)^c^	6330 (62.4)	3817 (39.1)	18,978 (52.1)	<0.001^d^
Parous	4523 (47.3)	3820 (37.6)	5937 (60.9)	17,460 (47.9)	
Smoking during pregnancy					
No	8286 (86.7)	9791 (96.5)	8832 (90.4)	33,386 (91.5)	<0.001^d^
Occasionally	369 (3.9)	144 (1.4)	254 (2.6)	980 (2.7)	
Daily	856 (9.0)	147 (1.4)	621 (6.4)	1926 (5.3)	
Missing data	51 (0.5)	68 (0.7)	60 (0.6)	199 (0.5)	
Alcohol during pregnancy					
No	8708 (91.1)	8682 (85.5)	8865 (90.9)	32,415 (89.0)	<0.001^d^
Yes	854 (8.9)	1468 (14.5)	889 (9.1)	4023(11.0)	
Maternal education (years)					
≤12	4069 (42.6)	1491 (14.7)	3547 (36.4)	11,337 (31.1)	<0.001^d^
13–16	3893 (40.7)	3901 (38.4)	4227 (43.3)	15,367 (42.2)	
≥17	1378 (14.5)	4554 (44.9)	1774 (18.8)	8966 (24.6)	
Missing data	222 (2.3)	204 (2.0)	206 (2.1)	768 (2.1)	
Household income (NOK)					
Both < 300,000	3202 (33.5)	1759 (17.3)	3227 (33.1)	10,135 (27.8)	<0.001^d^
Either ≥ 300,000	4087 (42.2)	3657 (36.0)	4197 (43.0)	15,074 (41.4)	
Both ≥ 300,000	1966 (20.6)	4521 (44.5)	1944 (19.9)	10,240 (28.1)	
Missing data	307 (3.2)	213 (2.1)	388 (4.0)	990 (2.7)	
Physical activity week 17^e^					
None	1988 (20.8)	746 (7.4)	1479 (15.2)	5127 (14.1)	<0.001^d^
<1/week	2214 (23.2)	1276 (12.6)	2021 (20.7)	7148 (19.6)	
1–2 times/week	2650 (27.7)	3020 (29.8)	2930 (30.0)	10,862 (29.8)	
≥3 times/week	1845 (19.3)	4482 (44.2)	2406 (24.7)	10,187 (28.0)	
Missing data	865 (9.0)	625 (6.2)	918 (9.4)	3112 (8.5)	
Nausea^f^					
Never	6100 (63.8)	7055 (69.5)	6401 (64.9)	24,075 (66.1)	<0.001^d^
Yes	3462 (36.2)	3095 (30.5)	3353 (35.1)	12,363 (33.9)	
Chronic diseases^g^					
No	9347 (97.8)	9953 (98.0)	9560 (98.0)	35,652 (98.8)	0.338^d^
Yes	215 (2.2)	197 (2.0)	194 (2.0)	786 (1.2)	
Eating disorder^h^					
No	9531 (99.7)	10,103 (99.6)	9742 (99.9)	36,312 (99.7)	<0.001^d^
Yes	32 (0.3)	44 (0.4)	12 (0.1)	124 (0.3)	

^a^Mean and standard deviation, all such numbers

^b^*p* value estimated with ANOVA

^c^Number of participants and percentage within that specific dietary group, all such numbers

^d^*p* value estimated with Pearson’s *χ*^2^ test

^e^Level of physical activity reported at gestational week 17

^f^Experience of nausea reported as never or only first trimester

^g^Chronic diseases (chronic hypertension, chronic kidney disease, systemisk lupus erythematosus, scleroderma, rheumatoid arthritis, or inflammatory bowl disease)

^h^Reported eating disorder during pregnancy

### Birthweight

Mean birth weight in the population was 3552 g, with an SD of 556 g. According to the ultrasound-based definition, 1324 (2.0%) of the babies were SGA and 2446 (3.7%) were LGA. According to the population-based definition, the corresponding respective figures were 5983 (9.1%) and 5797 (8.8%), while they were 9497 (14.4%) and 4485 (6.8%), respectively, according to the customized definition.

### Dietary patterns in relation to birth weight z-scores, SGA and LGA

**Fig. 1:**
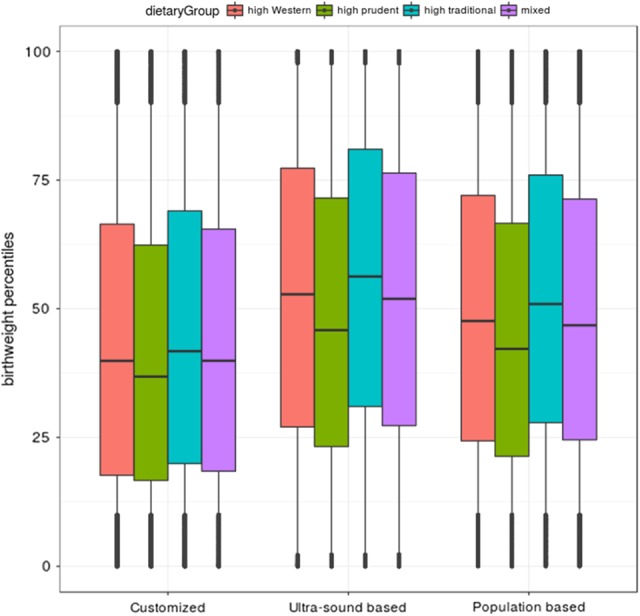
Boxplot of birth weight distribution within the four dietary pattern groups and according to the three different growth standards

Women in the high prudent group gave birth to infants with the lowest median birth weight percentile, unadjusted continuous data, according to all three growth standards. Infants born to women in the high traditional group had the highest median birth weight percentile according to all three growth standards, while the high Western and mixed group had quite similar birth weight distribution (Fig. 1).

In an adjusted linear regression, with the high Western group as reference, the high prudent group was significantly associated with lower birth weight *z*-scores, while the high traditional group was significantly associated with higher birth weight *z*-scores for all three growth standards (Table 3). There were no significant associations between birth weight *z*-scores for the mixed group compared to the high Western group. Additional adjustment for reported eating disorders and physical activity during pregnancy, did not change the results (results not shown).

**Table 3:** Infant birth weighs according to maternal dietary pattern in 65,904 mother–infant pairs in the Norwegian Mother and Child Cohort Study

Definition of birth weight	Dietary pattern	*β*-coefficient^a^	95% CI	*p* value^b^
Ultrasound-based	High Western	Reference	–	–
	High prudent	−0.041	−0.068, −0.013	0.004
	High traditional	0.067	0.040, 0.094	<0.001
	Mixed	0.013	−0.009, 0.034	0.247
Population-based	High Western	Reference	–	–
	High prudent	−0.034	−0.062, −0.007	0.014
	High traditional	0.068	0.041, 0.095	<0.001
	Mixed	0.013	−0.009, 0.034	0.250
Customized	High Western	Reference	–	
	High prudent	−0.038	−0.067, −0.009	0.010
	High traditional	0.009	0.039, 0.096	<0.001
	Mixed	0.015	−0.008, 0.037	0.208

^a^*β* = change in infant birth weight per unit of increased dietary pattern score, in relation to a high Western diet as reference

^b^*p* value according to linear regression adjusted for: maternal age, total energy intake, pre-pregnancy BMI, height, parity, smoking, alcohol intake, total household income, and maternal education

In agreement with the findings for the continuous birth weight data, women in the high prudent group had an increased risk of SGA and decreased risk of LGA (Table 4). This trend was seen across all three growth standards, but was only significant for SGA according to the ultrasound-based definition (adjusted OR 1.25, 95% CI: 1.02, 1.54) and for LGA according to the population-based and customized definitions (adjusted OR 0.84; 95% CI: 0.75, 0.94 and adjusted OR 0.88; 95% CI: 0.78, 0.99, respectively). The high traditional group was associated with reduced risk of SGA according to the customized definition (adjusted OR 0.92; 95% CI: 0.84, 0.99) and increased risk of LGA, significant according to the population-based and customized definitions: (adjusted OR 1.12; 95% CI: 1.02, 1.24 and adjusted OR 1.14; 95% CI: 1.03, 1.27, respectively). The mixed dietary group was not associated with the outcomes according to any of the definitions.

**Table 4:** Adjusted odds ratios (ORs) and 95% confidence intervals (95% CIs) for associations between dietary groups and SGA and LGA (three definitions of the outcomes) in 65,904 pregnant women in the Norwegian Mother and Child Cohort Study (MoBa)

Pattern	SGA, *N* (%)^a^	SGA adjusted, OR^b^ (95% CI)	LGA, *N* (%)	LGA adjusted, OR^b^ (95% CI)
Ultrasound-based				
High Western	177 (1.9)	1	378 (4.0)	1
High prudent	237 (2.3)	1.25 (1.02, 1.54)	262 (2.6)	0.87 (0.73, 1.02)
High traditional	186 (1.9)	1.16 (0.94, 1.43)	449 (4.6)	1.09 (0.94, 1.26)
Mixed	724 (2.0)	1.11 (0.94, 1.31)	1357 (3.7)	1.01 (0.89, 1.14)
Population-based				
High Western	889 (9.3)	1	891 (9.3)	1
High prudent	1087 (10.7)	1.04 (0.94, 1.15)	658 (6.5)	0.84 (0.75, 0.94)
High traditional	763 (7.8)	0.91 (0.82, 1.01)	1078 (11.1)	1.12 (1.02, 1.24)
Mixed	3244 (8.9)	0.94 (0.87, 1.02)	3170 (8.7)	0.97 (0.90, 1.05)
Customized				
High Western	1426 (14.9)	1	666 (7.0)	1
High prudent	1600 (15.8)	1.06 (0.98, 1.15)	563 (5.5)	0.88 (0.78, 0.99)
High traditional	1316 (13.5)	0.92 (0.84, 0.99)	781 (8.0)	1.14 (1.03, 1.27)
Mixed	5155 (14.1)	0.95 (0.89, 1.01)	2475 (6.8)	1.00 (0.92, 1.10)

^a^Number and percentage of cases in the dietary group

^b^Adjusted for maternal age, total energy intake, pre-pregnancy BMI, height, parity, smoking, alcohol intake, total household income, and maternal education

We repeated the analyses after stratification by pre-pregnancy BMI. Obesity is one of the strongest risk factors for having an LGA baby [43], but may also be associated with other adverse conditions during pregnancy, for example, hypertension, which may raise the risk of SGA [49]. In analyses stratified by BMI, comparable significant associations between the high prudent group and increased SGA risk and the traditional group and increased LGA risk were found in women with BMI <25 kg/m^2^ but not in the overweight group (Table 5).

**Table 5:** Adjusted odds ratios (ORs) and 95% confidence intervals (95% CIs) for associations between dietary groups and SGA and LGA (three definitions of the outcomes), stratified by maternal BMI in 65,904 pregnant women in Norwegian Mother and Child Cohort Study (MoBa)

Pattern	*N* (%)	SGA adjusted, OR^a^ (95% CI)	LGA adjusted, OR^a^ (95% CI)
BMI <25 kg/m^2^	46,077 (69.9)		
Ultrasound-based			
High Western		1	1
High prudent		1.28 (1.00, 1.64)	0.80 (0.63, 1.01)
High traditional		1.29 (1.00, 1.67)	1.14 (0.92, 1.41)
Mixed		1.19 (0.97, 1.47)	1.02 (0.85, 1.41)
Population-based			
High Western		1	1
High prudent		1.06 (0.94, 1.19)	0.73 (0.63, 0.85)
High traditional		0.90 (0.80, 1.02)	1.12 (0.98, 1.28)
Mixed		0.96 (0.87, 1.05)	0.91 (0.81, 1.01)
Customized			
High Western		1	1
High prudent		1.05 (0.95, 1.16)	0.89 (0.77, 1.03)
High traditional		0.95 (0.86, 1.05)	1.21 (1.06, 1.39)
Mixed		0.94 (0.87, 1.02)	1.01 (0.90, 1.13)
BMI ≥25 kg/m^2^	19,825 (30.1)		
Ultrasound-based			
High Western		1	1
High prudent		1.34 (0.87, 2.30)	1.09 (0.79, 1.49)
High traditional		0.88 (0.52, 1.50)	1.05 (0.80, 1.37)
Mixed		0.86 (0.58, 1.29)	0.96 (0.77, 1.20)
Population-based			
High Western		1	1
High prudent		1.13 (0.83, 1.52)	1.03 (0.87, 1.22)
High traditional		0.88 (0.66, 1.17)	1.13 (0.98, 1.31)
Mixed		0.87 (0.70, 1.08)	1.04 (0.93, 1.07)
Customized			
High Western		1	1
High prudent		1.10 (0.95, 1.27)	0.90 (0.73, 1.12)
High traditional		0.85 (0.74, 0.97)	1.06 (0.88, 1.27)
Mixed		0.96 (0.86, 1.06)	0.99 (0.86, 1.15)

^a^Adjusted for maternal age, total energy intake, height, parity, smoking, alcohol intake, total household income, and maternal education

We also wanted to profoundly assess associations between reported nausea during pregnancy in relation to SGA. Nausea during pregnancy leads to a limitation of food intake and increases the risk of SGA development [50]. It could be speculated that severe nausea during pregnancy could drive the women into a specific dietary pattern in order to try to ease nausea, but additional adjustment for this did not change the results.

Our results indicated that the high prudent group was associated with a higher risk of SGA. Women with eating disorders have lower BMI and significantly increased risk of having a SGA baby [51]. We re-ran the analysis after excluding women with eating disorders; however, that did not change the results. There was no significant difference in the number of women with eating disorders among the SGA cases and non-SGA cases, independent of SGA definition.

It could be hypothesized that women with serious underlying chronic disease choose to eat a healthy diet to prevent complications and that the association between the high prudent group and SGA might thus be confounded by the chronic disease. Additional adjustments for chronic diseases did not change the results (data not shown). Likewise, additional adjustment for maternal recreational physical activity did not influence the results (data not shown).

Finally, we repeated the analyses for term deliveries only (*n* = 62,415 mother–baby pairs) and found that the associations between the dietary groups and SGA were unchanged (data not shown).

Since certain macronutrients, vitamins, and minerals have been shown to be important in fetal growth [17], we performed an additional analysis comparing mean intakes of protein, vitamin D, calcium, folic acid, iron, and zinc in women with SGA infants compared to women with non-SGA infants within the high prudent group. We found that women eating according to the high prudent pattern giving birth to non-SGA babies had significantly higher intake of proteins, vitamin D, calcium, iron, and zinc. Mean daily differences were 2.2 ± 0.7 g for protein, 0.3 ± 0.1 µg for vitamin D, 13.9 ± 1.4 mg for calcium, 0.3 ± 0.2 g for iron, and 0.3 ± 0.0 mg for zinc. However, levels of folic acid were higher in women eating according to the high prudent pattern and giving birth to SGA babies, with a mean daily difference of 3.2 ± 3.4 µg.

## Discussion

### Main finding

In this study of 65,904 well-nourished Norwegian pregnant women, we found that compared to women with a high Western diet, women with a high prudent diet gave birth to babies with significantly lower birth weight and that women with a high traditional diet gave birth to babies with significantly higher birth weight. Moreover, being in the high prudent group was associated with an increased risk of SGA and decreased risk of LGA, in comparison with being in the high Western group. Contrarily, being in the high traditional diet group was associated with a decreased risk of SGA and increased risk of LGA, while the mixed group was not associated with differences in birth weight distribution or either SGA or LGA risk.

### Discussion of the findings

The findings for the association of maternal dietary quality and birth weight were consistent for all three growth standards applied. Compared to the high Western group the birth weight distribution was moved to lower values with higher SGA and lower LGA incidence for the high prudent group, while the whole birth weight distribution was moved to higher values with lower SGA and higher LGA incidence for the high traditional group. Even if analyses regarding SGA and LGA were not significant for all standards—the trend was the same. The cut-offs in birth weight definition of SGA and LGA vary with the ultrasound-based definition being the strictest and the customized based being the widest including most number of cases.

Thus, quality of maternal diet, regardless of total energy intake, might affect infant birth weight. It is important to point out that children born SGA do not have to be growth restricted, but might be healthy genetically small babies [52]. Also, maternal diet has been shown to correlate to body composition of the infants, rather than birth weight, for example, a high fat diet has been shown to be linked to increased neonatal adiposity [53], and a high prudent diet associated to lower neonatal adiposity [54].

When studying dietary patterns, the main focus is on diet quality, which is a result of the consumption pattern of all food items. Even if we cannot establish causality in this observational study, there are biological explanations for the findings that food quality can affect maternal metabolism and birth weight and thus SGA and LGA development. Women eating according to the high prudent dietary pattern had the lowest mean energy intake, and their food quality was characterized by the lowest amounts of total fat, carbohydrates, and added sugar, as well as the highest amount of dietary fibers, all of which could be correlated to lower risk of LGA. Dietary fiber has beneficial effects on blood glucose levels, as high intake counteracts the rise in postprandial plasma glucose following a meal and ensures that plasma glucose levels remain stable for a longer period of time [55, 56]. Lower plasma glucose levels are correlated to lower birth weight, even in women without diabetes mellitus [57]. A high intake of dietary fiber also increases satiety [58] and fiber intake has been associated with lower weight gain and lower risk of LGA in prospective epidemiological studies [59]. Among the overweight women in our study, we did not find a lower prevalence of LGA if they adhered to a high prudent diet. Obesity itself is one of the strongest risk factors for having an LGA baby [43] and it is believed that many of the negative obstetric outcomes seen in obese women are not preventable by lifestyle changes during pregnancy, as metabolic alterations are believed to occur prior to pregnancy [60]. The negative effects of obesity might thus exceed the positive effects of a high prudent diet during pregnancy in this regard. One explanation for the unexpected finding of increased SGA risk for babies to women in the high prudent group could be that some women in the high prudent group have extreme food intakes risking insufficient energy and nutrient supply to their babies. Mothers of SGA babies had significantly lower mean daily intake of energy, protein, vitamin D, calcium, iron, and zinc, all nutrients important for infant growth compared to non-SGA in the high prudent group.

The dietary characteristics for the high Western group are the opposite of those of the high prudent group, and it has previously been shown that high intake of dietary fat in early pregnancy is associated with increased birth weight and reduced risk of SGA [61]. Food with easily accessible carbohydrates increases postprandial plasma glucose levels [55], with subsequent increased risk of high birth weight [62].

Our results might have been confounded by women with underlying disease trying to eat as healthfully as possible in order to reduce the risk of complications. This possibility cannot be ruled out despite additional adjustments for some chronic diseases. We also hypothesized that eating disorders might have confounded the association between the high prudent group and increased risk of SGA, as women with anorexia nervosa are known to have an increased risk of SGA [51].

### Comparison with other studies

In contrast with our findings, in a Danish cohort of almost 45,000 women studied with a data-driven approach, Knudsen et al. [24] found that women with a high prudent diet (denoted “health conscious”) had a reduced risk of SGA according the same ultrasound-based definition as in our study, compared with women categorized as a Western dietary group. The health-conscious and Western groups had many of the same characteristics as the high prudent and high Western groups in our study. The health-conscious diet was characterized mainly by vegetables, fruit, poultry, and fish, while the Western pattern entailed high intake of red and processed meat. The Danish researchers defined a third “intermediate” group for foods that did not fit into either of the first two groups, while they did not identify a traditional dietary pattern. Therefore, the dietary exposure groups differed somewhat from the groups in our study, for example, while potatoes and margarine were included in the health-conscious food group, these food items belonged to the high traditional group in our study. Food intake and dietary patterns might differ even between countries as geographically close as Denmark and Norway; for instance, consumption of seafood and cod liver oil is higher in Norway [63]. A recently published article from China showed similar findings as the Danish study, and found that women adhering to a diet high in fruit, nuts, and desserts gave birth to infants with higher birth weight and lower prevalence of SGA [64]. In this study, the Guangzhou definition for SGA was used, which is similar to our customized definition. A third study which also contrasted with our results, involving 2000 pregnant women in New Zealand, showed that a traditional dietary pattern was associated with reduced risk of SGA. However, this pattern was rich in fruit and vegetables and was more similar to our high prudent group, but with a higher content of cheese and dairy products [65]. In another study within the MoBa cohort, it was shown that adherence to a New Nordic Diet, comparable to our high prudent group and characterized by Nordic fruit and vegetables, whole grains, potatoes, fish, milk, and water as beverage, was associated with lower weight gain during pregnancy as well as a reduced risk of SGA [66]. Finally, in a US study (*n* = 1 151) with seven different dietary patterns, no associations were reported between any of the patterns and SGA or LGA [67].

The comparison of different studies is complicated by strong correlations between nutritional habits, culture, and tradition. In addition to lifestyle factors, genetic factors strongly affect birth weight and lower comparability among different populations [14]. Different genotypes also have different risks of other diseases that could be related to adverse birth weight, for example, the increased incidence of gestational and type 2 diabetes among Asians [68]. Comparison between populations is therefore problematic. Furthermore, maternal characteristics in populations differ greatly; for example, BMI and maternal age affect epigenetics and genomic imprinting and the rates of subsequent development of adverse birth weight differ [69].

### Strengths and limitations

The major strength of this study is the large number of women, representing all parts of Norway and all socioeconomic groups. Another important strength is the prospective design; that is, dietary intake was assessed before the outcome was known. Using different definitions of SGA and LGA makes the results more comparable to those from other parts of the world. We also have information on a number of covariates, many of which have also been validated in other studies and shown to be accurate measures, for example, smoking [70]. Despite confounding factors having been taken into consideration, unidentified confounding may still have occurred. Furthermore, all dietary assessment methods have errors. FFQs are imprecise and prone to misreporting, as well as to errors related to portion size and nutrient calculation. However, the MoBa FFQ has been extensively validated and shown to be a valid tool for ranking pregnant women according to low and high intakes of energy, nutrients, and food items [29]. The participation rate in MoBa was 40%; MoBa women are generally healthier and better educated than the general population of pregnant Norwegian women [26]. However, Nilsen et al. [71] evaluated the potential bias due to self-selection to participate in the MoBa study, compared to the overall pregnant population in Norway, by evaluating exposure–outcome associations in MoBa and the MBRN. Despite the difference in prevalence of both exposures and outcomes between the two groups, there were no substantial differences in exposure–outcome associations, including for the outcome low birth weight.

## Conclusion

The prudent dietary pattern was associated with lower birth weight and the traditional dietary pattern was associated with higher birth weight in comparison with high adherence to the high Western diet. High adherence to the prudent pattern was associated with increased risk of SGA and reduced risk of LGA. Conversely, high adherence to the traditional dietary pattern was associated with lower risk of SGA and higher risk of LGA. Our results suggest that, in addition to food quantity, food quality may have an effect on birth weight.

## Electronic supplementary material

Supplementary Table S1

## Data Availability

The dataset used in this study originates from the Norwegian Mother–Child cohort study (MoBa), regulated by the MoBa Scientific Management Group. All data used from MoBa are subject to legal restrictions prohibiting the authors from making minimal data sets publicly available. For further information contact: datatilgang@fhi.no, or Professor Per Magnus (per.magnus@fhi.no).
