# Supplementary material for: Associations between maternal dietary patterns and infant birth weight, small and large for gestational age in the Norwegian Mother and Child Cohort Study
Source: Eur J Clin Nutr. 2018 Nov 20;73(9):1270–82. doi: 10.1038/s41430-018-0356-y (PMC6760641; doi:10.1038/s41430-018-0356-y)
Supplement: Supplementary file 1 — Supplementary Table S1 [file 41430_2018_356_MOESM1_ESM.docx]

### Supplementary Table S1. Daily intake of food items in the four dietary groups

| **Food group** | **High Western** | **High prudent** | **High traditional** | **Mixed** |
| --- | --- | --- | --- | --- |
| White bread^1^ | 159±98^2^ | 52±68 | 118±103 | 111±100 |
| Dark bread | 44±72 | 120±97 | 106±110 | 90±99 |
| Butter | 2.8±7.4 | 3.4±7.6 | 3.2±8.0 | 3.4±8.2 |
| Margarine | 12±13 | 6.0±9.6 | 15.6±15 | 12±14 |
| Cheese | 20±18 | 25±19 | 24±20 | 23±20 |
| Fish spread | 7.6±9.8 | 12±13 | 12±16 | 11±14 |
| Fish liver/roe | 0.1±0.9 | 0.2±1.0 | 0.6±3.1 | 0.4±2.3 |
| Meat spread | 22±19 | 19±17 | 25±21 | 23±20 |
| Mayonnaise/spread | 14±13 | 10±9.5 | 11±11 | 13±13 |
| Jam/honey | 8.1±12 | 8.2±12 | 11±15 | 9.4±14 |
| Nutspread | 5.1±12 | 1.1±3.5 | 2.8±7.5 | 2.8±7.8 |
| Eggs | 10±12 | 14±15 | 9.3±9.8 | 11±13 |
| Cereals, high grain | 14±24 | 44±58 | 22±33 | 26±44 |
| Corn flakes | 2.7±7.1 | 1.1±4.7 | 2.0±5.6 | 2.1±6.4 |
| Full fat milk | 48±128 | 23.5±72 | 41±123 | 42±123 |
| Low fat milk | 274±295 | 219±243 | 404±357 | 303±316 |
| Yoghurt/biola | 67±102 | 139±170 | 71±100 | 97±139 |
| Juice | 177±196 | 182±177 | 146±172 | 178±197 |
| Artificially sweetened drinks | 261±447 | 86±191 | 78±182 | 131±267 |
| Sugar drinks | 289±400 | 60±107 | 140±202 | 152±154 |
| Coffee | 81±152 | 113±146 | 100±169 | 103±168 |
| Decaffeinated coffee | 2.6±27 | 7.3±41 | 2.0±22 | 3.5±29 |
| Tea | 92±156 | 142±199 | 112±175 | 124±189 |
| Herbal tea | 20±64 | 118±219 | 33±90 | 54±141 |
| Water for drinking | 969±681 | 1500±700 | 1103±656 | 1198±725 |
| Processed meat | 94±30 | 55±27 | 68±25 | 73±29 |
| Red meat | 18±11 | 14±10 | 15±9.8 | 16±11 |
| Organ meat | 0.1±0.7 | 0.1±1.1 | 0.4±1.9 | 0.3±1.6 |
| Poultry | 20±13 | 31±17 | 13±9.8 | 21±15 |
| Lean fish | 3.6±4.4 | 8.8±7.3 | 13±11 | 7.8±8.0 |
| Oily fish | 2.6±3.5 | 6.4±6.1 | 5.5±5.8 | 4.8±5.3 |
| Fish products | 12±10 | 17±11 | 33±18 | 20±15 |
| Pizza/tacos | 26±12 | 17±9.1 | 15±8.6 | 20±10 |
| Boiled potatoes | 37±23 | 29±20 | 65±29 | 44±31 |
| French fries | 12±10 | 5.1±4.9 | 6.1±5.1 | 7.9±8.2 |
| Rice | 26±22 | 36.4±29 | 18±12 | 27±24 |
| Pasta/spaghetti | 26±20 | 24±18 | 16±11 | 22±18 |
| Ketchup | 2.5±2.7 | 1.1±1.2 | 1.3±1.3 | 1.6±1.9 |
| Olive oil | 0.6±0.8 | 1.4±1.1 | 0.5±0.7 | 0.8±0.9 |
| Cooking oil | 0.9±1.5 | 4.2±5.4 | 0.7±1.1 | 1.7±3.1 |
| Raw vegetables other than leafy greens | 16±16 | 41±32 | 18±17 | 26±28 |
| Green leafy vegetables | 56±43 | 104±59 | 53±40 | 72±55 |
| Cooked vegetables | 34±23 | 62±40 | 45±29 | 50±43 |
| Onion/leek/garlic | 7.5±7.3 | 15±12 | 5.6±5.4 | 9.5±10 |
| Mushroom | 3.0±3.6 | 6.1±6.7 | 2.3±2.4 | 4.0±5.7 |
| Gravy | 15±11 | 8.3±6.9 | 18±14 | 15±14 |
| Fruit/berries | 223±163 | 351±206 | 241±161 | 285±215 |
| Dried fruits | 1.3±3.2 | 6.7±13 | 2.1±5.1 | 3.2±2.5 |
| Nuts | 1.4±2.8 | 5.3±12 | 1.2±2.5 | 2.3±6.0 |
| Milk desserts | 24±22 | 18±15 | 15±12 | 21±21 |
| Waffles/pancakes | 12±9.2 | 8.3±6.6 | 11±7.6 | 11±10 |
| Buns | 7.4±11 | 4.9±5.6 | 4.2±4.8 | 5.9±8.6 |
| Cakes | 9.9±8.3 | 7.2±5.9 | 6.9±5.5 | 8.9±9.8 |
| Chocolate sweets | 67±52 | 37±27 | 32±24 | 45±39 |
| Salty snacks | 20±16 | 11±8.6 | 11±7.8 | 14±14 |
| Rice pudding | 6.5±7.6 | 4.5±6.7 | 12±11 | 7.5±8.5 |
| Crisp bread | 11±16 | 16±19 | 13±18 | 15±19 |
| Sweet biscuits | 4.0±8.0 | 2.6±4.6 | 2.1±3.5 | 3.3±6.9 |

^1^ Grams per day

^2^ Mean and standard deviation
